# An Experimental Study on Sarcasm Comprehension in School Children: The Possible Role of Contextual, Linguistics and Meta-Representative Factors

**DOI:** 10.3390/brainsci13060863

**Published:** 2023-05-26

**Authors:** Rachele Fanari, Sergio Melogno, Roberta Fadda

**Affiliations:** 1 Department of Pedagogy, Psychology, Philosophy, University of Cagliari, 09126 Cagliari, Italy; 2 Department of Psychology of Development and Socialization Processes, “Sapienza” University of Rome, 00185 Rome, Italy; 3 Faculty of Psychology, “Niccolò Cusano” University of Rome, 00166 Rome, Italy

**Keywords:** sarcasm, school children, Theory of Mind, contextual factors

## Abstract

Understanding sarcasm is a complex ability, which includes several processes. Previous studies demonstrated the possible roles of linguistic and meta-representative factors in understanding sarcasm in school children, while the influence of specific contextual variables still needs to be investigated. Here, we present two studies investigating the possible role of contextual, linguistics, and meta-representative factors in understanding sarcasm in school children. In Study 1, we investigated sarcasm comprehension in 8–9-year-old school children in three different contexts, in which both familiarity and authority were manipulated. We found that understanding sarcasm was facilitated when the conversational partner was characterized by a high level of authority and familiarity (the mother) rather than when the conversational partner was an adult with a lower level of both authority and familiarity (the cashier of a food store). In Study 2, we replicated and extended Study 1 by investigating the possible influence of the same contextual factors but in a more sizeable sample and at different ages: first, third, and fifth grades of primary school. We found that understanding sarcasm improved significantly with age. The results of both studies indicated that understanding sarcasm is influenced by contextual factors. Children at any age better understood sarcasm produced by a speaker with a high level of both familiarity and authority. This ability improved with age. These results expand our understanding of how children infer a speaker’s intentions in sarcasm. This might be particularly of interest to develop possible interventions for children on the Autism Spectrum, who are known to misunderstand sarcasm at different levels of complexity.

## 1. Introduction

Verbal irony is a figure of speech in which the implicit meaning is the opposite of the apparent meaning [1,2]. Sarcasm is a type of counterfactual verbal irony characterized by several distinctive characteristics. First, sarcasm is directed at a specific person. Second, compared to other forms of irony, such as hyperbole, understatement, and rhetorical questions [3], sarcasm is characterized by a more direct discrepancy between the uttered statement and the intended message, a more pronounced use of humor and ridicule, and is usually more aggressive and offensive [4,5,6]. The main communicative goals of sarcastic irony are to mock or tease, to reprimand or indirectly criticize someone, and to be funny or witty [7]. Usually, people use sarcasm to express judgment toward a negative behavior. Basically, by using sarcasm, people praise a behavior to express, indeed, a negative judgment. For example, when faced with a completely burned roast chicken, a person might express a sarcastic comment such as “Congratulations, perfect cooking!” Sarcasm has been extensively investigated in adults. 

Understanding sarcasm is a complex ability that includes several processes. It requires inferring the communicative intention of the speaker [8]. Additionally, it involves the ability to detect the mismatch between a negative property of a behavior or an event (i.e., a burned food) vs. the positive statement about the person involved in the scene (i.e., “You are really a good cooker!”). It is also important to know whether a social norm has been violated [9]. Finally, it is crucial to understand whether and to what extent the speaker shares listener-specific contextual information, which defines the actual meaning of a sarcastic sentence [10].

A traditional account proposed by Grice [11] suggests a pragmatic view of verbal irony, in which the understanding of the ‘figurative meaning’ of a sentence is triggered by a violation of the first quality maxim “Do not say what you believe to be false” [11]. However, this traditional account does not explain why, in different cultures, if someone says the opposite of what he/she means, the implication of the utterance will be perceived as pleasing by others. Thus, according to the traditional account, verbal irony utterances are more difficult to process than their literal meaning, but it does not determine any extra benefit. This might render the use of verbal irony irrational and effortful. 

Another traditional account, the ‘literal-first’ model [3], states that the figurative interpretations of verbal irony are harder to construct than literal interpretations. So, it takes more effort and more time for the listener to understand the figurative meaning of the sentence than the literal one. 

To overcome the limits of the traditional accounts, Sperber and Wilson [12] proposed the echoic account, according to which the speaker of an ironical utterance is not saying the opposite of what she means but echoing a thought she attributes to others and expressing a mocking attitude to this thought. An evolution of Sperber and Wilson’s account is the pretense theory [1], extended by Kumon-Nakamura et al. [13]. According to pretense theories, the speaker of an ironical utterance is merely pretending to perform a speech act, while expecting her audience to see through the pretense and detect the mocking attitude behind it.

These perspectives properly describe adults’ understanding of verbal irony, with a marked focus on the role of meta-representational abilities and counterfactual thinking. Few developmental investigations have specifically tested children’s processing of non-literal speech-act. 

From a developmental perspective, direct forms of verbal irony, such as sarcasm, are understood earlier in development than other forms of irony. This might be due to the pronounced and thus easily detectable discrepancy between the uttered statement and the intended message [14]. Typically developing children understand ironic utterance early in development if it refers to a contextual cue that might be perceived in the environment, rather than when it refers to an imaginary shared knowledge between the speakers. Angeleri and Airenti (2014) [15] found that 3- and 4-year-old children showed a good understanding of the actual intent of an ironic communicative act when the utterance implied the negation of something directly perceived by the interlocutor. Children detect verbal irony without perceived contextual cues at approximately the age of 5 or 6 [16,17,18,19]. Appreciation of verbal irony continues to develop through school years, between the ages of 7 and 10, with a clear understanding of the speaker’s communicative purpose [9,20,21]. Understanding sarcasm is generally set in adolescents and adults [22].

Investigations in children might inform researchers interested in pragmatic language development by pointing to new views of verbal irony understanding. It seems that both meta-representational and pragmatics abilities emerging early on in development might play a key role in children’s understanding of verbal irony, including sarcasm.

Some studies indicated a relationship between irony and sarcasm comprehension and Theory of Mind abilities [8,9,19,23]. 

A study on school children investigated sarcasm comprehension in three groups of Italian children at 6, 8, and 10 years of age [9], in relation to two different contexts: parent–child interaction and sibling interaction. The results indicated that sarcasm was better understood when the speaker was the mother rather than a sibling. There was also a correlation between understanding sarcasm and Theory of Mind abilities. 

However, other research did not find any association between Theory of Mind abilities and irony [15,24]. Additionally, Zajaczkowska and Abbot-Smith [25] examined the ability to interpret simple and complex irony in two studies—respectively with 6- to 8-year-old children and 10- to 12-year-old children. Researchers controlled for non-verbal reasoning, structural language, and specific knowledge. The results of both studies indicated that only cognitive flexibility but not Theory of Mind abilities contributed to complex irony comprehension. 

Another promising line of research considers the contextual factors that might play a key role in irony and understanding sarcasm. Specifically, some studies indicated that speaker–addresser relationship cues might foster sarcasm comprehension by facilitating the meta-representational processing involved in understanding irony [26]. 

Whalen and collaborators [27] showed a group of children aged 5 to 8 years old several stories that ended with an ironic criticism and involved pairs of speakers who were either siblings or people who had just met. The results showed that knowledge about the relationship between the speakers did not affect the correctness of recognizing sarcastic intent. However, this knowledge reduced the reaction time in deciding if the final statement was sarcastic or not. The authors concluded that speaker–addresser relationship cues should be considered relevant in any model that describes irony processing [28].

Massaro et al. [9] found that authority influences sarcasm comprehension in school children, by presenting a series of open-ended stories in which characters with high vs. low levels of authority—respectively, the mother and the siblings—produced a sarcastic statement. Participants better understood the sarcastic statements produced by the mother. Additionally, Theory of Mind abilities played a key role in understanding sarcasm. Both the relationships considered in this study were close relationships, characterized by a high level of familiarity. The shared common ground between people in a close relationship might facilitate the meta-representational inferences involved in sarcasm comprehension.

However, different types of close relationships might give different clues for understanding sarcasm. The mother–child relationship is hierarchical while the sibling relationship is more reciprocal. Massaro et al. [9] demonstrated that different types of close relationships might provide clues about the speaker’s attitude in sarcasm comprehension, at least at the earlier stage of processing.

This leaves open the question of whether familiarity might influence sarcasm comprehension in school children. 

Our study aimed to answer this question by investigating the role of two contextual factors—respectively the hierarchical nature of the relationship and familiarity—in understanding sarcasm. We replicated and extended Massaro et al.’s [9] work. We presented a series of open-ended stories in which a character produced a sarcastic statement. We manipulated both the hierarchical nature of the relationship and familiarity of the character in three kinds of relationships: a mother–child relationship, a sibling relationship, and a stranger–child relationship. At the end of the stories, participants had to indicate the character’s intention (being sarcastic or not) and his/her belief (thinking the opposite of what is said or not). We also evaluated participants’ non-verbal cognitive performance and Theory of Mind abilities. 

We predicted that participants would better understand the sarcastic statement produced by characters they have a hierarchical relationship with. We also predicted that participants would better understand sarcasm produced by the character with a higher level of familiarity.

## 2. Method

### 2.1. Study 1

In Study 1, we investigated sarcasm comprehension in school children in three different contexts, in which both familiarity and the hierarchical nature of the relationship were manipulated. We hypothesized that understanding sarcasm might be facilitated when the conversational partner is characterized by the hierarchical nature of the relationship and familiarity (i.e., the mother) rather than when the conversational partner is an adult with a lower hierarchical nature of the relationship and familiarity (i.e., the cashier of a food store). We also predicted that understanding sarcasm in the sibling relationship would lie between these two conditions we just described. We also explored the possible correlation between sarcasm comprehension, general cognitive abilities, and Theory of Mind abilities. 

### 2.2. The Participants

A group of 55 children in the fourth grade of a primary school (30 males; 25 females), with a mean age of 115.51 months (SD = ±2.530), participated in this study. We decided to investigate children at this age because a previous study indicated that children begin to understand the intended humor in irony at age nine or ten [28]. Additionally, since girls seem to better understand sarcasm than boys, we controlled for this effect by investigating sarcasm in a sample equally distributed between girls and boys [29].

Informed consent was obtained from the parents of all the participants involved in this study, and the local ethics committee granted its approval for this study.

### 2.3. The Procedure

Participants were evaluated individually in a quiet room at school. We evaluated general cognitive abilities with the Progressive Colored Matrices [30] and digit span forward and backward [31]. We also administered the second-order false belief test, the ”Ice-cream van” task [32] and a series of Irony Tasks.

#### 2.3.1. The Theory of Mind Task

We evaluated participants’ Theory of Mind abilities with a second-order false-belief story, the "Ice cream van” test [32]. Two characters (John and Mary) were independently informed about an ice cream van’s unexpected transfer to a new location. Both John and Mary knew where the van was but John thought that Mary thought that the van was still in its original place. Participants’ understanding of the second-order belief was tested by asking them where John thought Mary would go for ice cream. Correct answers could only be given if John’s second-order belief was represented.

#### 2.3.2. Digit Span Forward and Backward

Participants were asked to repeat forward and backward a string of numerals spoken by the examiner. The string was made progressively longer to determine the numerals that could be recalled. 

#### 2.3.3. Raven’s Progressive Matrices 

Raven’s Progressive Matrices (often referred to simply as Raven’s Matrices) are multiple-choice intelligence tests of abstract reasoning [32]. Participants are asked to identify the missing item that completes a pattern, presented in the form of a 4 × 4, 3 × 3, or 2 × 2 matrix.

#### 2.3.4. Irony Tasks

To evaluate sarcasm comprehension, the experimenter presented three tasks of sarcasm comprehension, which we developed for this study, in line with those used by Massaro et al. [9] and Filippova and Astington [8]. The tasks differed for two dimensions related to speaker status (familiarity and authority), as follows:

(a) A parent–child relationship (high level of authority/high level of familiarity),
(b) A sibling relationship (same level of authority/high level of familiarity), and
(c) An occasional relationship between a stranger adult and a child—the cashier at the supermarket (low level of authority/low level of familiarity).

The three stories are described below:

 **(a)**  **Parent–child story**

*“Francesco is at home and it’s dinner time. Francesco is playing in his room while his mother is cooking. When the dinner is ready, the mother calls Francesco and she asks him to set the table for dinner. Francesco puts everything on the table but in a chaotic way, so the mother says to him “Oh, well, you are definitely precise in setting the table!”.*

**Question to investigate the children’s understanding of the belief of the mother:** When the mother says to Francesco “Oh, well, you are definitely precise in setting the table!”, does she think that Francesco is precise or messy?

 **(b)**  **Sibling story**

*“Alessio decides to fix the wheel of his bicycle in collaboration with his brother. Alessio swear to his brother that, once they fixed the wheel, they would put away together all the tools that they used into a box. However, once the wheel was fixed, Alessio takes the bicycle and goes for a ride, without honoring his promise about putting away the tools. So, his brother says “Oh, well, you are really organized in putting away the tools!”*.

**Question to investigate the children’s understanding of the belief of the mother:** When the sibling says to Alessio “Oh, well, you are really organized in putting away the tools!”, does he think that Francesco is organized or messy?

 **(c)**  **A stranger adult interacting with a child—the cashier story**

*“Laura goes to the supermarket with her mother. They buy a lot of food. When they arrive at the cashier’s desk, they check whether they missed anything. In doing so, they slowly put the items on the desk. The cashier looks at them and says: “Wow, you guys are really fast!”.*

**Question to investigate the children’s understanding of the belief of the cashier**: 

When the cashier says to Laura “Wow, you guys are really fast!”, does she think that Laura is fast or slow?

### 2.4. Coding of the Irony Tasks

We considered “correct” all the answers to the belief questions in which the participants understood the belief of the speaker, namely the opposite of what the protagonist literally says. Correct answers to the stories were scored with 1 point. We considered “incorrect” the answers to belief questions in which the participants did not understand the speaker’s belief, namely the opposite of what the protagonist says. The incorrect answers to the stories were scored with 0 points.

### 2.5. Results

We evaluated a series of cognitive abilities that might be important for irony comprehension (Table 1).

We also considered possible gender differences in understanding sarcasm. The results indicated that boys better understood sarcasm in the sibling story compared to girls (Mann–Whitney test = 315; *p* < 0.001).

**Figure 1:**
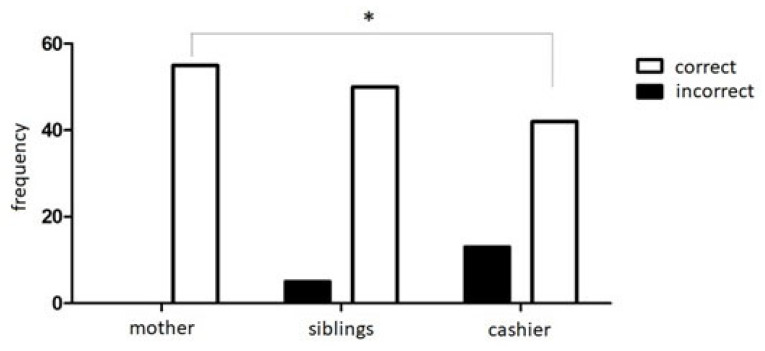
Frequency of correct and incorrect responses to the three stories of sarcasm comprehension. Asterisks indicate the significant differences at α ≤ 0.05.

We compared the participants’ correct answers to the belief questions in the three sarcasm stories. As shown in Figure 1, the Q Cochran test for repeated measures indicated significant differences between the stories (χ2 = 16.125; df = 2; *p* < 0.001). Specifically, the McNemar test for repeated measures indicated that participants better understood sarcasm in the story with the mother compared to the story with the stranger adult (*p* < 0.001).

The Fisher test did not indicate significant differences between the children that pass or fail the second-order false belief test (Table 2) in the understanding of sarcasm in the three stories involving, respectively, the mother, the siblings, and the cashier. 

There were no significant correlations between understanding sarcasm and the scores obtained by the participants in Raven’s Matrix and the digit span forward and backward.

In Study 1, we found that children better understand sarcasm produced by the mothers compared to sarcasm produced by a stranger adult. We did not find any influence of Theory of Mind abilities and general cognitive abilities in understanding sarcasm. Thus, contextual factors such as familiarity and the hierarchical nature of the relationship seem to play a major role in understanding sarcasm. However, in this study, the sarcasm produced by the cashier was directed both to the mother and to the child at the same time. This might render the story with the stranger–child interaction difficult to compare with the other two stories—respectively, that with the mother–child interaction and the sibling interaction. To overcome this methodological limitation, we developed Study 2, in which we modified the story with the stranger–child relationship so that the sarcasm was directed only at the child. Additionally, we considered a more sizeable sample and children of different ages to see whether understanding sarcasm improves with age. 

## 3. Study 2: Understanding Sarcasm at Different Ages

In Study 2, we replicated and extended Study 1 by investigating the possible influence of contextual factors in a more sizeable sample and at different ages. We also modified the story with the stranger–child relationship so that the sarcasm was directed only at the child. We expected that understanding sarcasm would improve significantly with age, thanks to a better understanding of the pragmatic implication of the different conversational contexts [10]. We also investigated the possible relation between understanding sarcasm and general language abilities, other than Theory of Mind abilities as in Study 1. 

### 3.1. Participants

A sample of 180 school children (90 males; 90 females) took part in this study: 61 children (29 males; 32 females) were in the first grade (mean age = 81.30 ± 3.685); 60 children (29 males; 31 females) were in the third grade (mean age = 104.27 ± 3.804); 59 children (32 males; 27 females) were in the fifth grade (mean age = 128.31 ± 3.626). Informed consent was obtained from the parents of all the participants involved in this study, and the local ethics committee granted its approval for this study. None of the participants in Study 2 also participated in Study 1.

### 3.2. Procedure

Participants were evaluated individually in a quiet room at school. We evaluated general cognitive abilities with the Progressive Colored Matrices [30], the digit span forward and backward [31], and a series of language comprehension tasks from the TROG test [31]. We also administered the second-order false belief test, the "Ice cream van” test [32]. To evaluate sarcasm comprehension, the experimenter presented the same tasks of sarcasm comprehension of Study 1, the parent–child story, and the sibling story, except for the cashier story, which has been modified. 

#### 3.2.1. The TROG Test—The Test for Reception of Grammar

The Test for Reception of Grammar is a multiple-choice test where the child listens to a spoken sentence and must select one of four pictures to match what is heard. We used a short version adapted for Italian children from 5 to 11 years of age [31].

#### 3.2.2. Irony Tasks

The cashier story was modified as follows:

*“Laura goes to the supermarket with her mother. They buy a lot of food. When they arrive at the cashier’s desk, Laura helps the mother put the item on it. In doing so, she was very slow. The cashier looks at her and says: “Wow, you are really fast!”.*

**Question to investigate the children’s understanding of the belief of the cashier:** When the cashier says to Laura “Wow, you are really fast!”, does she think that Laura is fast or slow?”

### 3.3. Coding of the Irony Tasks

As in Study 1, we considered “correct” all the answers to the belief questions in which the participants understood the belief of the speaker, namely the opposite of what the protagonist literally says. Correct answers to the stories were scored with 1 point. We considered “incorrect” the answers to belief questions in which the participants did not understand the speaker’s belief, namely the opposite of what the protagonist says. The incorrect answers to the stories were scored with 0 points.

### 3.4. Results

We evaluated the participants in a series of measures that describe their cognitive functioning (Table 3). 

**Figure 2:**
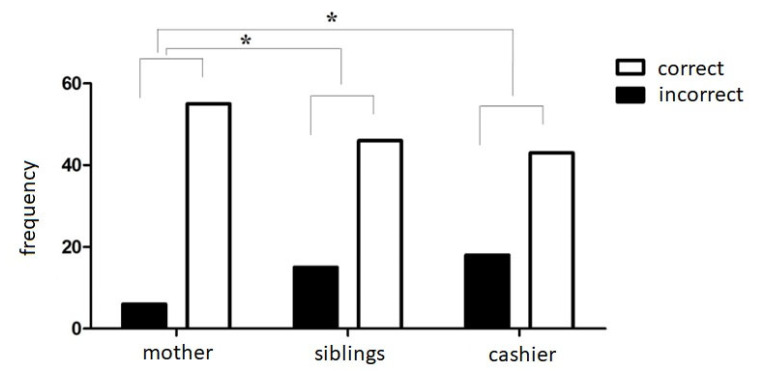
Frequency of correct and incorrect responses to the three stories of sarcasm comprehension in the participants attending the first grade of school. Asterisks indicate the significant differences at α ≤ 0.05.

We compared the participants’ correct answers to the belief questions in the three sarcasm stories. As shown in Figure 2, the Q Cochran test for repeated measures indicated significant differences between the stories (χ2 = 12.316; df = 2; *p* ≤ 0.002) in the participants who attended the first grade. Specifically, the McNemar test for repeated measures indicated that participants better understood sarcasm in the story with the mother compared to the story with the cashier (*p* = 0.002). Additionally, participants better understood sarcasm in the story with the mother rather than with the siblings (*p* = 0.012).

The Fisher test did not indicate significant differences between the children that pass or fail the second-order false belief test in the understanding of sarcasm in the three stories involving, respectively, the mother, the siblings, and the cashier at any of the different ages considered. Additionally, we did not find any significant gender effect in the parent–child story (F = 2.159; *p* = 0.145; µ^2^ = 0.023), in the sibling story (F = 0.148; *p* = 0.702; µ^2^ = 0.002) and in the cashier story (F = 0.050; *p* = 0.823; µ^2^ = 0.001).

There were significant differences according to age only for the understanding of sarcasm in the relationship with the cashier of the supermarket, which improved significantly with age (Kruskal–Wallis = 13,563; df = 2; *p* = 0.001). 

There were no significant correlations between understanding sarcasm and the scores obtained by the participants in Raven’s Matrices and the digit span forward and backward. 

In the children attending the fifth grade, there was a significant correlation between understanding sarcasm in the story with the TROG lexical scores (r = 0.341; *p* = 0.008), the TROG syntactic scores (r = 0.274; *p* = 0.036) and the TROG total scores (r = 0.269; *p* = 0.040). 

## 4. Discussion

Our studies investigated understanding sarcasm from a developmental perspective. We considered both the role of meta-representational abilities related to mental states and the contextual factors as possible cues to process sarcasm in children.

Previous studies indicated a key role of Theory of Mind and socio-communicative abilities [8]. Additionally, contextual factors seem to play a key role. Massaro et al. [9] found that authority influences sarcasm comprehension in school children, by presenting a series of open-ended stories in which characters with high vs. low levels of authority—respectively the mother and the siblings—produced a sarcastic statement. Participants better understood the sarcastic statements produced by the mother. Theory of Mind played a key role in understanding sarcasm. However, the character presented in Massaro et al.’s [9] study did not differ in terms of familiarity, which was very high in both cases. This leaves open the question of whether familiarity might influence sarcasm comprehension in school children. 

We aimed to answer this question by investigating the role of two contextual factors—respectively the hierarchical nature of the relationship and familiarity—in understanding sarcasm. 

We set two studies, by replicating and extending Massaro et al.’s [9] work. We presented a series of open-ended stories in which a character produced a sarcastic statement. We manipulated both the hierarchical nature of the relationship and familiarity of the character in three kinds of relationships: a mother–child relationship, a sibling relationship, and a stranger–child relationship. We also evaluated participants’ non-verbal cognitive performance and Theory of Mind abilities. We predicted that in line with Massaro et al. [9], participants will better understand the sarcastic statement produced by characters with a high hierarchical nature of the relationship. We also predicted that participants would better understand sarcasm produced by the character with a higher level of familiarity.

In Study 1, we found that children better understand sarcasm produced by mothers (which is characterized by both the hierarchical nature of the relationship and familiarity), compared to sarcasm produced by a stranger adult, such as the cashier of a supermarket (which is characterized by a low hierarchical nature of the relationship and familiarity). We did not find any influence of Theory of Mind abilities and general cognitive abilities in understanding sarcasm. Thus, we can conclude that specific contextual factors such as those we investigated, namely familiarity and the hierarchical nature of the relationship, might play a major role in understanding sarcasm. It might be that specific pragmatic abilities are involved, such as the expectations that a child has in relation to a specific character in a different context about sarcasm production. Children might consider adults as expert communicators, who can produce sarcasm. However, being an adult might be a necessary condition but not a sufficient one, since the sarcasm produced by an unfamiliar adult might not be understood as well as the that produced by the mother. These results are in line with Recchia et al. [33], indicating that children show an understanding of ironic language, especially sarcasm, in the context of naturalistic family conversations at home. Mothers are especially likely to ask rhetorical questions and use ironic language in conflictual contexts. So, it might be that children are more familiar with sarcasm produced by the mother compared to a stranger.

However, an alternative explanation of our results might be that, in Study 1, the sarcasm produced by the cashier was directed both to the mother and to the child at the same time. This might have been a methodological limitation of our Irony Tasks, which might render the story with the stranger–child interaction difficult to compare with the other two stories—respectively the one with mother–child interaction and the sibling interaction.

Based on this consideration, we developed Study 2, in which we adjusted the cashier story so that the adult only addressed the child. We also considered different ages to explore possible differences in sarcasm comprehension. The results indicated that children in the first grade understood sarcasm better when it was produced by the mother rather than by the siblings or the cashier. However, this phenomenon seems to disappear in older children. These results are only apparently in contrast with those of Study 1, since we modified the story of the cashier in study two, letting her address only the child and not both an adult and a child. So, in this case, children seem to better understand sarcasm from a stranger as they grow older. These results seem to indicate that being an adult is not a sufficient condition to understand sarcasm but that children evaluate the general interactional context: who produces the sarcasm and who is being addressed by the sarcasm. This study might confirm what was indicated in previous studies—that children might base their sarcasm on understanding implicit pragmatic norms [9]. However, differently from Massaro et al. [9], we did not find any relation between Theory of Mind abilities and sarcasm. Since we used only one Theory of Mind task, we might have underestimated possible individual differences between participants in this ability. This might be considered a possible limitation of this study. In a future study, we might use a battery of Theory of Mind tests, which might allow us to overcome a dichotomic measure of Theory of Mind abilities (presence/absence). In Study 2, we found an interesting correlation between linguistic abilities and sarcasm comprehension. This correlation needs to be better explored, maybe considering a more sizeable sample at different ages. This would allow us to identify possible linguistic sub-phenotypes, which might correlate with understanding sarcasm. 

Another possible future line of research might be to consider understanding sarcasm in other relational scenarios, in which the level of familiarity and authority might vary in other ways. For example, we might investigate sarcasm comprehension in the mother of the child’s best friend. In this direction, we could also pair, in future studies, possible standardized measures of pragmatic abilities and figurative language, to better define the possible individual differences in pragmatic abilities underlying sarcasm. 

Finally, a further possible future line of research might be to consider the role of individual differences in understanding sarcasm in childhood. Rothermich et al. [31] invited a large sample of school children aged between 8 and 12 years old to watch video clips of young adults using different speaker intentions. The results also suggest that children already show adult-like abilities in understanding literal statements at the age of 8 years, whereas the ability to infer specific social intentions increases between the ages of 8 and 12 years. Moreover, girls performed better in classifying sarcasm than boys. In our study, we found that males outperformed girls in understanding sarcasm. New studies are needed to better understand the nature of these results. In Study 2, we confirmed that understanding sarcasm improved with age. However, in Study 1, we found that males outperformed girls in understanding sarcasm while we did not find any gender effect in Study 2. Thus, more studies are needed to better explore the role of gender in understanding sarcasm in childhood.

## 5. Conclusions

Understanding sarcasm is a complex ability, which includes the ability to detect the speaker’s intention and to differentiate it from the literal meaning of the utterance. However, the role of meta-representative factors in understanding sarcasm in school children is not straightforward. Additionally, contextual factors seem to play an important role. 

Here, we investigated the possible role of both mentalistic abilities and contextual factors in sarcasm comprehension among school children. The results of our studies indicate that children at any age better understand sarcasm produced by a speaker with a high level of familiarity and with a hierarchical relationship. This ability improves with age. 

Given that we did not find a relationship between Theory of Mind and sarcasm comprehension, we suggest that the role of contextual factors may be a promising line of research to understand how children learn to interpret sarcasm. Another possible future line of research might investigate sarcasm comprehension among children on the Autism Spectrum, who are known to naturally lack Theory of Mind abilities. This investigation might help to clarify the role of both mentalistic abilities and contextual factors in understanding sarcasm. 

## Figures and Tables

**Table 1:** Number, mean, and standard deviation of participants in the following measures: digit span forward and backward, and Progressive Colored Matrices.

Test	N	Mean	SD
Digit Span Forward	55	5.18	0.84
Digit Span Backward	55	3.91	0.89
Progressive Colored Matrices	55	103.3	11.1

**Table 2:** Frequencies of correct and incorrect answers to the three stories of sarcasm comprehension (mother, siblings, and cashier) in relation to correct and incorrect responses in the second-order false belief test. We did not find any significant difference at α ≤ 0.05.

Stories		Second-Order False Belief		Fisher Test
		Correct	Incorrect	
mother	correct	0	0	*p* = 0.546
	incorrect	21	34	
sibling	correct	2	11	*p* = 0.1
	incorrect	19	23	
cashier	correct	2	3	*p* = 1
	incorrect	19	31	

**Table 3:** Number, mean, and standard deviation of participants in the first, third, and fifth grades in the following measures: digit span forward and backward, Progressive Colored Matrices, TROG, and Peabody.

Test	School Grade	N	Mean	SD
Digit Span Forward	First grade	61	4.38	0.877
	Third grade	60	5.22	1.083
	Fifth grade	59	6.15	0.944
Digit Span Backward	First grade	61	2.52	0.595
	Third grade	60	3.62	1.091
	Fifth grade	59	4.47	1.023
Progressive Colored Matrices	First grade	61	106.2	12.0
	Third grade	60	105.5	13.3
	Fifth grade	59	106.4	13.1
TROG	First grade	61	104.2	12.8
	Third grade	60	104.9	13.2
	Fifth grade	59	104.7	15.0

## Data Availability

The data presented in this study are available on request from the corresponding author.
